# Advice to Women

**Published:** 1891-02

**Authors:** 


ADVICE TO WOMEN, respecting some of the ailments peculiar to their sex. By J. Adams, M. D., M. C. P., and S. D., Toronto, Canada. Roswell & Hutchinson, Printers, 1890.

In this little brochure of some eighty pages, Dr. Adams calls attention in vigorous terms to the current methods of the old school in its treatment of the diseases of women. It is an interesting volume, and gives a note of timely warning against a barbarous practice; for the current methods of the so-called gynæcologists are simply barbarous butchery. Dr. Adams also shows, in contrast to this treatment, as Drs. Guernsey, Skinner, and others have done, how readily these female complaints yield to true homœopathic treatment. Rest in bed, with judicious use of hot water, may be used in conjunction with homœopathic medicines. E. J. L.

